# Short-term safety outcomes of mastectomy and immediate prepectoral implant-based breast reconstruction: Pre-BRA prospective multicentre cohort study

**DOI:** 10.1093/bjs/znac077

**Published:** 2022-04-05

**Authors:** Kate L Harvey, Parisa Sinai, Nicola Mills, Paul White, Christopher Holcombe, Shelley Potter, Pre-BRA Feasibility Study Steering Group, Peter Barry, Rachel O'Connell, Simon Cawthorn, Matthew Gardiner, Gareth Irwin, Cliona Kirwan, Mairead McKenzie, Shireen McKenzie, Georgette Oni, Lisa Whisker, Tim Rattay, Pankaj Roy, Joanna Skillman, Soni Soumian, Raghavan Vidya, Samantha Williams

**Affiliations:** National Institute for Health Research Bristol Biomedical Research Centre, University Hospitals Bristol NHS Foundation Trust and University of Bristol, Bristol, UK; National Institute for Health Research Bristol Biomedical Research Centre, University Hospitals Bristol NHS Foundation Trust and University of Bristol, Bristol, UK; National Institute for Health Research Bristol Biomedical Research Centre, University Hospitals Bristol NHS Foundation Trust and University of Bristol, Bristol, UK; Applied Statistics Group, University of the West of England, Bristol, UK; Breast Unit, Royal Liverpool University Hospital, Liverpool, UK; National Institute for Health Research Bristol Biomedical Research Centre, University Hospitals Bristol NHS Foundation Trust and University of Bristol, Bristol, UK; Bristol Breast Care Centre, North Bristol NHS Trust, Bristol, UK

## Abstract

**Background:**

Prepectoral breast reconstruction (PPBR) has recently been introduced to reduce postoperative pain and improve cosmetic outcomes in women having implant-based procedures. High-quality evidence to support the practice of PPBR, however, is lacking. Pre-BRA is an IDEAL stage 2a/2b study that aimed to establish the safety, effectiveness, and stability of PPBR before definitive evaluation in an RCT. The short-term safety endpoints at 3 months after surgery are reported here.

**Methods:**

Consecutive patients electing to undergo immediate PPBR at participating UK centres between July 2019 and December 2020 were invited to participate. Demographic, operative, oncology, and complication data were collected. The primary outcome was implant loss at 3 months. Other outcomes of interest included readmission, reoperation, and infection.

**Results:**

Some 347 women underwent 424 immediate implant-based reconstructions at 40 centres. Most were single-stage direct-to-implant (357, 84.2 per cent) biological mesh-assisted (341, 80.4 per cent) procedures. Conversion to subpectoral reconstruction was necessary in four patients (0.9 per cent) owing to poor skin-flap quality. Of the 343 women who underwent PPBR, 144 (42.0 per cent) experienced at least one postoperative complication. Implant loss occurred in 28 women (8.2 per cent), 67 (19.5 per cent) experienced an infection, 60 (17.5 per cent) were readmitted for a complication, and 55 (16.0 per cent) required reoperation within 3 months of reconstruction.

**Conclusion:**

Complication rates following PPBR are high and implant loss is comparable to that associated with subpectoral mesh-assisted implant-based techniques. These findings support the need for a well-designed RCT comparing prepectoral and subpectoral reconstruction to establish best practice for implant-based breast reconstruction.

## Teaser

Prepectoral breast reconstruction is gaining in popularity, but evidence to support its safety and effectiveness is lacking. This prospective multicentre cohort study of 347 women and 424 reconstructions suggests that complication rates are high at 3 months after surgery, with 8.2 per cent of women experiencing implant loss, 19.5 per cent requiring treatment for an infection, and 16.0 per cent requiring further surgery for complications. These complication rates are comparable to those seen in subpectoral mesh-assisted reconstruction, and this study strongly supports the need for a well designed randomized trial comparing prepectoral and subpectoral implant-based reconstruction techniques.

## Introduction

Despite improvements in breast cancer treatment, up to 40 per cent^1^ of the 55 000^2^ women diagnosed with breast cancer each year in the UK require a mastectomy. Immediate breast reconstruction is offered routinely to improve quality of life^3^.

Implant-based reconstruction is the most commonly performed breast reconstruction procedure worldwide^4,5^. This was initially a two-stage technique with the insertion of a tissue expander under the pectoralis major muscle; multiple expansions were undertaken until the desired volume was achieved, followed by a second procedure to replace the expander with a definitive fixed-volume implant. The introduction of biological and synthetic mesh approximately 10 years ago offered the potential for single-stage direct-to-implant reconstruction without the need for time-consuming and uncomfortable expansions^6^. The use of mesh as a sling between the lower pole of the pectoralis muscle and the chest wall also improved the cosmetic outcome of implant-based procedures by allowing greater lower pole projection, and broadened the indications for implant-based techniques^7^. Despite the lack of high-quality evidence to support the benefits of mesh-assisted subpectoral procedures^8–12^, they rapidly became established as standard of care in the UK^12^.

Recently, mesh-assisted implant-based reconstruction has evolved further, with the implant, wrapped fully or partially in mesh, placed on top, rather than under the muscle^13^. This muscle-sparing or prepectoral technique may reduce postoperative pain and avoids implant animation, the upward movement of an implant seen when the pectoralis muscle contracts^14^. Prepectoral implant placement may also reduce the incidence of capsular contracture following postmastectomy radiotherapy^15^ and avoid chronic pain associated with raising the muscle^16^.

High-quality comparative evidence to support the benefits of prepectoral reconstruction over subpectoral mesh-assisted techniques is limited^17^. Subcutaneous implant placement was previously abandoned by the reconstructive community because of unacceptably high complication rates^18–21^. The most recent systematic review^22^ summarized 15 studies comparing prepectoral and subpectoral implant-based reconstruction. The authors undertook a meta-analysis and concluded that the techniques were broadly equivalent, with similar rates of complications, implant loss, and patient-reported outcomes. There was some limited evidence to suggest a lower risk of capsular contracture in the prepectoral group. Many of the studies included were, however, small, heterogeneous, and often retrospective single-centre studies with limited follow-up. Since the publication of this review, at least 25 additional studies have been published comparing complications^12–38^, process outcomes such as expansion volumes and analgesic use^16–42^, patient-reported outcomes^16,26,33,34,37,41,43^ or, less frequently, cosmetic outcomes^16,24,38^ of prepectoral and subpectoral techniques. Overall, the results favour prepectoral reconstruction, but these are non-randomized mostly retrospective single-centre studies at high risk of bias. Furthermore, many of the studies report outcomes from expert North American centres using two-stage expander–implant reconstruction, which does not reflect UK practice.

There is a need for a well designed pragmatic RCT to compare the clinical and cost-effectiveness of prepectoral and subpectoral implant-based breast reconstruction, but RCTs in breast reconstruction are challenging^44,45^. As prepectoral techniques have been introduced recently, it is not clear whether they are safe or sufficiently stable for definitive evaluation in a trial. The IDEAL (Idea, Development, Exploration, Assessment, Long-term study) Framework provides recommendations for the evaluation of a surgical innovation from first-in-man to long-term study^46,47^. This article reports the safety outcomes of an IDEAL 2a/2b study that aimed to evaluate the safety, effectiveness, and stability of prepectoral implant-based reconstruction before definitive evaluation in an RCT.

## Methods

### Study design and participants

Pre-BRA was a single-arm, multicentre, IDEAL stage 2a/2b prospective observational cohort study with embedded qualitative methods^48^ (registration number ISRCTN11898000). This paper reports the 3-month safety outcomes. The 18-month effectiveness and stability outcomes will be reported elsewhere.

All UK breast and plastic surgical centres performing prepectoral breast reconstruction using any technique were invited to participate in the study.

Women aged 16 years or over requiring a mastectomy for breast cancer or risk reduction, who elected to undergo immediate implant-based breast reconstruction, and were considered technically suitable for prepectoral reconstruction by the surgeon, were eligible for inclusion. Women undergoing delayed reconstruction or revisional procedures, those considered to have insufficient soft tissue coverage for prepectoral reconstruction, and those not willing or able to give informed consent were excluded. For surgeons experienced in undertaking prepectoral reconstruction, no further exclusion criteria were applied as a key objective of the study was to capture current practice. For surgeons with less experience, or those starting to offer the technique, caution was recommended in groups of patients likely to be at higher risk of developing complications. Relative exclusion criteria were based on Association of Breast Surgery and British Association of Plastic, Reconstructive and Aesthetic Surgeons guidelines for mesh-assisted breast reconstruction^49^, and included: current smokers or recent ex-smokers; women with a BMI of more than 30 kg/m^2^; diabetics; those who had received previous breast or chest wall radiotherapy or who were likely to require radiotherapy after surgery; and women in whom the implant volume was anticipated to be greater than 600 ml^49^. Surgeons were asked to risk stratify patients before surgery based on these factors. Women with no risk factors were considered at low risk of complications; those with one risk factor were considered at moderate risk, and those with two or more risk factors were considered at high risk of developing postoperative complications. Operative teams were also asked to report whether patients were considered to be high risk for other reasons.

Full ethical approval was obtained for the study (NRES OXFORD-B South Central Committee Ref:19/SC/0129; IRAS ID: 255421) and women provided fully informed written consent before study entry.

### Procedures

Eligible patients were identified prospectively from multidisciplinary meetings, clinics, and operating lists at participating centres. The study was introduced at a clinic consultation and eligible patients were given a study information sheet. All eligible patients were followed up by a member of the clinical or research team and asked to provide written consent before participating in the study.

All patients were given an operation date in accordance with local unit protocol, and simple demographic, co-morbidity, and operative data were collected for each participant via a standardized electronic case report form (CRF) hosted on REDCap^50^. Participants were asked to complete baseline patient-reported outcome questionnaires (BREAST-Q) and have preoperative photographs before surgery, in line with unit policy.

All patients had a skin or nipple-sparing or a skin-reducing mastectomy, and immediate prepectoral implant reconstruction with or without mesh was planned for each patient at the point of study entry. Any mesh with a CE mark licensed for use in the UK could be used in the study, but the choice of mesh (biological or synthetic, and product used) and implant selection (fixed volume; adjustable implants or tissue expanders) were according to surgeon preference. Composite prepectoral reconstructions using a dermal sling and mesh, or two different types of mesh were permitted.

Participating surgeons undertook the procedure according to their standard practice but, for the purposes of ensuring procedure fidelity, insertion of a tissue expander or implant was considered a mandatory step, and raising pectoralis major muscle was prohibited when performing the procedure^51^. All other steps were based on the operating surgeon’s preference.

If, during the procedure, the operating surgeon considered that prepectoral reconstruction was not possible owing to concerns about skin-flap viability or because the preoperative plan was modified (for example, insertion of a tissue expander rather than the planned fixed-volume implant), this was recorded with details of why a change was needed and the alternative procedure performed (such as subpectoral reconstruction).

Strategies to minimize infection (for example, use of laminar flow, cavity irrigation, glove change) were implemented in accordance with local practice, but participating centres were encouraged to adhere to published best practice guidelines^49,52^ and use the evidence-based Manchester checklist^53^, which was included as part of the electronic CRF. Drains and other concomitant interventions, such as antibiotics and dressings, were permitted in line with local practice.

Complications and oncological data were collected at 30 days and 3 months by clinical or case-note review according to local follow-up policies. No additional clinic visits were required for the study. All complications were defined *a priori* using standardized definitions as in previous studies^12,54^, and the recently developed core measurement set for implant-based breast reconstruction^55^. Participants were asked to complete patient-reported outcome questionnaires either by post or online including a visual analogue pain score at 24 h, 1 week, 2 weeks, and 3 months after surgery, and the BREAST-Q (version 2)^56^ reconstruction module and the newly developed animation subscale at 3 and 18 months after surgery.

### Outcome measures

The primary outcome for the safety study was implant loss at 3 months, defined by removal of the implant or tissue expander without replacement owing to a postoperative complication. This is consistent with the definition used in the iBRA study^12^. Secondary safety outcomes were complications requiring readmission or reoperation and infection requiring treatment at 3 months. Infections were considered minor if oral antibiotics only were required, and major if readmission was needed for intravenous antibiotics and/or surgical debridement.

### Statistical analysis

A single-arm design was used to assess the safety of prepectoral implant reconstruction based on the primary outcome of implant loss at 3 months. It was anticipated that the implant loss rate would be 9 per cent or lower based on findings from the recent iBRA study^12^. A sample size of 310 patients would result in a two-sided 95 per cent confidence interval for a single proportion, assumed to be 0.10, with a width equal to 0.070. Allowing for 10 per cent loss to follow-up at 3 months, it was calculated that at least 341 patients were needed to inform a future trial with implant loss as the primary outcome.

Simple summary statistics were calculated to describe demographic, procedure, process, and outcome data. Categorical data are summarized as counts and percentages and continuous data as median (i.q.r.; range). The percentage, with 95 per cent confidence interval, of patients experiencing implant loss and other key safety outcomes (readmission for complications, reoperation, and infection) at 3 months was calculated, and compared with those reported in the iBRA study^12^. Univariable logistic regression was used to undertake an exploratory risk factor analysis to explore patient- and procedure-related variables that were hypothesized, based on published literature, to influence complication rates following implant-based reconstruction. Risk factors considered included: age; BMI (less than *versus* 30 or more kg/m^2^); smoking (current smoker, recent (stopped at diagnosis) ex-smoker, those on nicotine replacement *versus* non-smokers, long-term ex-smokers); bilateral surgery (yes *versus* no); indication (at least one malignancy *versus* risk reduction); previous breast/chest wall radiotherapy (yes *versus* no); neoadjuvant chemotherapy (yes *versus* no); duration of operation; type of mesh (biological *versus* synthetic); mastectomy weight (specimen weight less than 600 *versus* 600 g or more); surgeon’s intraoperative assessment of mastectomy skin-flap viability (good *versus* average or poor); type of mastectomy (at least one nipple-sparing procedure *versus* other types of mastectomy); anticipated implant volume exceeding 600 ml (yes *versus* no); and surgeon’s preoperative assessment of patient risk (low *versus* moderate, high) based on the number of preoperative risk factors identified.

## Results

**Fig. 1:**
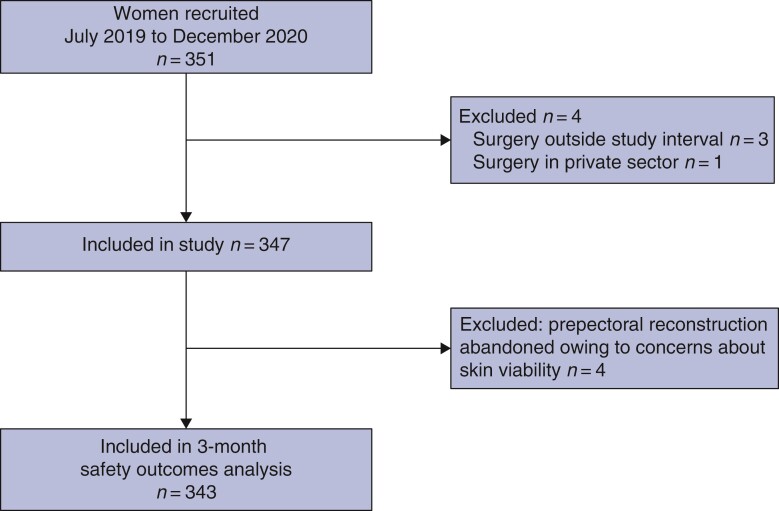
Pre-BRA study flow diagram

A total of 351 patients were recruited from 40 participating UK centres between 1 July 2019 and 31 December 2020. This included an unplanned pause to recruitment between March and July 2020 during the initial alert phase of the COVID-19 pandemic when breast reconstruction was halted to allow prioritization of emergency care^57^. Of the 351 patients recruited to the study, three had surgery outside the study interval and one underwent surgery in the private sector, and were therefore withdrawn. Some 347 patients were included in the analysis (*Fig. 1*).

Patient demographics are summarized in *Table 1* and *2*. Almost half of all participants (163, 47.0 per cent) were overweight or obese, and 41 (11.8 per cent) were current smokers, recent ex-smokers (stopped at diagnosis) or used nicotine replacement. Only 25 women (7.2 per cent) had undergone previous chest wall or mantle radiotherapy; 147 (42.4 per cent) were either possibly or probably likely to require radiotherapy after mastectomy. Implant volumes of more than 600 ml were predicted in only 20 patients (5.8 per cent). Overall, based on surgeons’ preoperative assessment of risk of postoperative complications, 211 women (60.8 per cent) had no risk factors and were considered low risk, 103 (29.7 per cent) had one risk factor and were considered moderate risk, and 16 (4.6 per cent) had more than one risk factor and were considered high risk; a further four patients were considered to be at high risk of postoperative problems for another reason (*Table 2*). Patients recruited during the COVID-19 pandemic (July to December 2020) were more likely to be considered low risk (no preoperative risk factors) than those recruited earlier in the study (data not shown).

**Table 1:** Patient demographics

	No. of patients* (*n* = 347)
**Age (years)**†	49 (41–56; 23–74)
**BMI (kg/m^2^)**†	24.6 (22.5–28.7; 17.7–42.8)
Underweight (< 18.5)	3 (0.9)
Normal (18.5–24.9)	172 (49.6)
Overweight (25.0–29.9)	93 (26.8)
Obese (> 30.0)	70 (20.2)
Not reported	9 (2.6)
**Smoking status**	
Non-smoker	303 (87.3)
Current smoker	8 (2.3)
Nicotine replacement/vaping with nicotine	11 (3.1)
Recent ex-smoker (stopped at diagnosis)	22 (6.3)
Not reported	3 (0.9)
**Indication for surgery**	
Malignancy	259 (74.6)
Risk reduction	42 (12.1)
Malignancy/risk reduction	43 (12.4)
Not reported	3 (0.9)
**Laterality of surgery**	
Unilateral	262 (75.5)
Bilateral	84 (24.2)
Not reported	1 (0.3)
**Co-morbidities**	
Yes	85 (24.5)
No	251 (72.3)
Not reported	11 (3.2)
**Diabetes**	4 (1.2)
**Recruitment period**	
Before COVID-19 pandemic	223 (64.3)
During COVID-19 pandemic	124 (35.7)
**ASA grade**	
I	198 (57.1)
II	134 (38.6)
III	10 (2.9)
IV	1 (0.3)
Not reported	4 (1.2)
**Previous radiotherapy to ipsilateral breast**	
Yes	25 (7.2)
No	311 (89.6)
Not reported	11 (3.2)
**Neoadjuvant chemotherapy**	
Yes	49 (14.1)
No	245 (70.6)
Not reported	53 (15.3)
**Previous surgery to ipsilateral breast**	
Yes	91 (26.2)
No	252 (72.6)
Not reported	4 (1.2)
**Type of previous surgery**	
Axillary surgery	38 (11.0)
Wide local excision	63 (18.2)
Augmentation	8 (2.3)
Reduction	2 (0.6)
Other	13 (3.8)

**Table 2:** Patient demographics

	No. of patients* (*n* = 347)
**Surgeon’s assessment of suitability for prepectoral reconstruction and consideration of contraindications**	
Previous radiotherapy to ipsilateral breast‡	25 (7.2)
Surgeon’s assessment of likely requirement for postmastectomy radiotherapy‡	
Not required (previous risk-reducing surgery or radiotherapy)	41 (11.8)
Unlikely	90 (25.9)
Possible	104 (37.4)
Probable	43 (15.5)
Not reported	69 (19.9)
Ptosis	
None	76 (21.9)
Grade 1 (mild)	100 (28.8)
Grade 2 (moderate)	82 (23.6)
Grade 3 (advanced)	57 (16.4)
Grade 4 (severe)	5 (1.4)
Not reported	27 (7.8)
Current smoking/vaping or nicotine replacement or recent ex-smoker (stopped at diagnosis)‡	41 (11.8)
Predicted implant volume ≥ 600 ml‡	20 (5.8)
BMI ≥ 30 kg/m^2^‡	70 (20.2)
**Overall surgeons’ perceived potential risk of complications**‡	
Low risk (no relative CI)	211 (60.8)
Moderate risk (1 relative CI)	103 (29.7)
High risk (> 1 relative CI)	16 (4.6)
High risk for another reason	4 (1.2)
Not reported	13 (3.7)

*With percentages in percentages unless indicated otherwise; †values are median (i.q.r.; range). ‡Relative contraindications (CIs): previous breast/mantle radiotherapy; anticipated postmastectomy radiotherapy; BMI over 30 kg/m^2^; current smoker; implant volume exceeding 600 ml.

The 347 women underwent 424 breast reconstruction procedures (*Table 3*). Operations were mainly performed by consultant surgeons (362, 85.4 per cent), the majority of whom had considerable experience with the technique; 189 (44.6 per cent) had undertaken over 25 operations unsupervised (*Table S1*). Nipple-sparing mastectomies were performed in over half of the patients (221, 52.1 per cent), with inframammary fold incisions used in 119 (28.1 per cent). The median mastectomy weight was 410 (i.q.r. 263–590; range 49–2009) g. The procedure was carried out as planned in 411 of 424 instances (96.9 per cent), mainly using biological mesh (341, 80.4 per cent) as a single-stage with a fixed-volume implant (334, 78.8 per cent). The median implant volume was 395 (300–480; 125–825) ml. Prepectoral reconstruction was abandoned in four women (1.2 per cent) because of concerns about skin-flap viability (*Fig. 1*). The procedure was converted to subpectoral implant-based reconstruction in all four women, two with and two without the use of mesh. The median duration of surgery was 165.5 (130–190.5; 60–420) min. Almost all women received antibiotics at induction (342, 98.6 per cent) but other infection prevention measures, such as double gloving and pocket washing, were used variably (*Table S2*). More than half of patients (201, 57.9 per cent) required an overnight hospital stay; only a small number (50, 14.4 per cent) had day-case procedures (*Table S1*).

**Table 3:** Oncology data for patients having mastectomy for malignancy and multidisciplinary team decision-making for adjuvant therapies

	No. of patients*
**Oncology data per breast**	*n* = 309
Invasive disease	212 (68.6)
Ductal carcinoma *in situ*	60 (19.4)
Not reported	37 (12.0)
Multifocal disease	93 (30.1)
Tumour grade	
Grade 1 (low)	21 (6.8)
Grade 2 (intermediate)	111 (35.9)
Grade 3 (high)	126 (40.8)
Not reported	51 (16.5)
Node-positive (*n* = 247)	63 (25.6)
No. of involved nodes†	1 (1–4; 1–22)
Size of invasive disease (mm)†	22 (12–37; 0–150)
**MDT recommendations (per patient)**	*n* = 300
Adjuvant chemotherapy	69 (23.0)
Radiotherapy	82 (27.3)
Endocrine therapy	185 (61.7)
HER2 treatment	43 (14.3)
No. of patients requiring adjuvant chemotherapy and/or radiotherapy‡	110 (36.7)
Interval from last cancer surgery to first adjuvant treatment†	53.5 (41–71; n.a.)

*With percentages in percentages unless indicated otherwise; †values are median (i.q.r.; range). ‡Patients may be recommended chemotherapy or radiotherapy or both treatments by the multidisplinary team (MDT). HER2, human epidermal growth factor receptor 2; n.a., data not available.

*Table 3* shows the oncological data for 300 women who underwent 309 mastectomies for malignancy. Surgery was mainly for invasive disease (212, 68.6 per cent); one in four women had at least one involved axillary node (63 of 246, 25.6 per cent). Adjuvant chemotherapy (69, 23.0 per cent) and/or radiotherapy (82, 27.3 per cent) was recommended in 110 women (36.7 per cent), and the median time from last surgery to first adjuvant treatment in this group was 53.5 (i.q.r. 41–71) days.

Of the 343 women who underwent prepectoral implant-based reconstruction (*Fig. 1*), 144 (42.0 (95 per cent c.i. 36.7 to 47.2) per cent) experienced at least one postoperative complication (*Table 4*). Sixty-seven patients (19.5 (15.3 to 23.8) per cent) developed an infection, 60 (17.5 (13.5 to 21.5) per cent) required readmission for complications, and 55 (16.0 (12.1 to 19.9) per cent) underwent further surgery for complications within 3 months of the initial reconstruction. Twenty-eight women (8.2 (5.3 to 11.1) per cent) experienced implant loss and reconstruction failure during this interval; a further 11 (3.2 per cent) had a successful implant salvage procedure. Further details of complications are shown in *Table S3*.

**Table 4:** Three-month outcomes after prepectoral breast reconstruction compared with outcomes in the iBRA study

	Pre-BRA study (*n* = 343)	iBRA study (*n* = 2081)
**Readmission**	60 (17.5; 13.5, 21.5)	372 (18; 16–20)
**Reoperation**	55 (16.0; 12.1, 19.9)	372 (18; 16–20)
**Infection**	67 (19.5; 15.3, 23.8)	522 (25; 23–27)
Implant loss*	28 (8.2; 5.3, 11.1)	182 (9; 8, 10)
**Any complication**	144 (42.0; 36.7, 47.2)	

Values in parentheses are percentages with 95 per cent confidence intervals. *Total removal of implant/expander without replacement. There were 11 additional patients in whom successful implant salvage (with a tissue expander or implant) was performed. Salvage was unsuccessful in one; this is included as a total implant loss at 3 months.

Exploratory risk factor analysis identified that having radiotherapy previously (odds ratio (OR) 4.53, 95 per cent c.i. 1.63 to 12.61; *P* = 0.004), a mastectomy weight of more than 600 g (OR 2.34, 1.01 to 5.41; *P* = 0.047), and being considered at moderate or high risk before operation (OR 3.58, 1.54 to 8.31; *P* = 0.003) were strongly associated with increased odds of experiencing implant loss, whereas smoking or nicotine use was associated with readmission (OR 2.49, 1.20 to 5.15; *P* = 0.014), reoperation (OR 2.18, 1.02 to 4.68; *P* = 0.045), and infection (OR 2.41, 1.18 to 4.90; *P* = 0.015) at 3 months (*Table 5*). Surgeons’ intraoperative assessment that mastectomy skin-flap viability was average or poor was strongly associated with an increased odds of requiring both readmission (OR 2.07, 1.11 to 3.86; *P* = 0.023) and reoperation (OR 2.18, 1.15 to 4.14; *P* = 0.017). Reoperation was also associated with increasing age (OR 1.04, 1.01 to 1.07; *P* = 0.010) and mastectomy weight of more than 600 g (OR 2.01, 1.05 to 3.88; *P* = 0.036). Neither obesity (BMI exceeding 30 kg/m^2^) nor duration of operation was associated with complications in this exploratory analysis.

**Table 5:** Exploratory univariable logistic regression of risk factors for complications

	Implant loss		Readmission		Reoperation		Infection	
	Odds ratio	*P*	Odds ratio	*P*	Odds ratio	*P*	Odds ratio	*P*
**Age (per year)**	1.04 (1.00, 1.07)	0.053	1.02 (1.00, 1.05)	0.090	1.04 (1.01, 1.07)	0.010	1.02 (0.99, 1.04)	0.229
**Obesity (BMI ≥ 30 kg/m^2^)**	1.03 (0.40, 2.65)	0.949	1.05 (0.53, 2.08)	0.882	0.93 (0.45, 1.92)	0.849	0.89 (0.45, 1.74)	0.727
**Current smoking/nicotine use**	1.75 (0.62, 4.90)	0.288	2.49 (1.20, 5.15)	0.014	2.18 (1.02, 4.68)	0.045	2.41 (1.18, 4.90)	0.015
**Previous radiotherapy**	4.53 (1.63, 12.61)	0.004	1.29 (0.46, 3.58)	0.632	2.26 (0.89, 5.73)	0.085	2.16 (0.89, 5.25)	0.090
**Neoadjuvant chemotherapy**	1.33 (0.47, 3.76)	0.586	0.47 (0.18, 1.25)	0.132	0.56 (0.21, 1.49)	0.244	0.73 (0.32, 1.66)	0.452
**Bilateral surgery**	0.65 (0.24, 1.76)	0.393	0.64 (0.32, 1.30)	0.220	0.64 (0.31, 1.33)	0.233	1.06 (0.57, 1.95)	0.863
**At least one mastectomy for malignancy**	0.83 (0.27, 2.51)	0.741	1.32 (0.53, 3.30)	0.549	0.96 (0.40, 2.28)	0.919	1.04 (0.46, 2.37)	0.917
**Biological mesh**	2.62 (0.34, 20.03)	0.354	3.19 (0.74, 13.8)	0.121	1.26 (0.42, 3.77)	0.681	0.74 (0.08, 7.28)	0.799
**Duration of operation (per min)**	1.00 (0.99, 1.01)	0.629	1.00 (1.00, 1.01)	0.666	1.00 (0.99, 1.01)	0.846	1.00 (0.99, 1.00)	0.500
**Anticipated large implant (≥ 600 ml)**	3.20 (0.98, 10.48)	0.055	1.71 (0.59, 4.95)	0.321	2.32 (0.85, 6.38)	0.102	2.56 (0.97, 6.75)	0.058
**At least one nipple-sparing procedure (*versus* other mastectomy patterns)**	1.77 (0.77, 4.10)	0.181	1.13 (0.64, 1.99)	0.674	1.21 (0.69, 2.18)	0.532	1.24 (0.72, 2.13)	0.444
**Good skin-flap vascularity (*versus* average or poor)**	2.07 (0.88, 4.88)	0.094	2.07 (1.11, 3.86)	0.023	2.18 (1.15, 4.14)	0.017	1.25 (0.66, 2.37)	0.486
**Mastectomy weight ≤ 600 g (*versus* > 600 g)**	2.34 (1.01, 5.41)	0.047	1.37 (0.71, 2.65)	0.350	2.01 (1.05, 3.88)	0.036	1.55 (0.83, 2.89)	0.170
Considered as being at moderate/high risk of complications before surgery*	3.58 (1.54, 8.31)	0.003	1.05 (0.59, 1.89)	0.867	1.82 (1.00, 3.28)	0.049	0.95 (0.54, 1.67)	0.849

Values in parentheses are 95 per cent confidence intervals. *At least one risk factor from: high BMI, current smoking, previous radiotherapy, anticipated postmastectomy radiotherapy, anticipated implant volume exceeding 600 ml; or considered high risk by operating surgeon for another reason.

## Discussion

This prospective multicentre study evaluated the short-term safety of prepectoral breast reconstruction. It included 424 reconstructions in 347 women across 40 UK centres, and provides high-quality real-world data regarding the practice and outcomes of prepectoral breast reconstruction in the UK. Although the majority of procedures were performed in women who were fit and well with a low risk of complications, one-third were undertaken in those with at least one established risk factor and a small proportion in women considered to be at high risk of experiencing a postoperative complication. Most procedures were single-stage direct-to-implant reconstructions with biological mesh, often following nipple-sparing mastectomies, and almost all planned prepectoral reconstructions were completed successfully with a few converted to subpectoral techniques owing to skin-flap viability concerns. Overall rates of postoperative complications, however, were high, with over 40 per cent of women experiencing at least one postoperative complication. Implant loss was reported in 8.2 per cent of patients; readmission and reoperation were reported in 17.5 and 16.0 per cent respectively. Almost one in five women experienced a postoperative infection requiring treatment.

Exploratory risk factor analysis identified smoking/nicotine use as being associated with readmission, reoperation, and infection, whereas previous radiotherapy, mastectomy weight of more than 600 g, and the surgeon’s preoperative assessment of the patient being at moderate or high risk of complications were associated with implant loss. The operating surgeon’s intraoperative assessment of average or poor mastectomy skin-flap viability was associated with both readmission and reoperation for complications at 3 months. In contrast to the findings of the iBRA study^12^, however, neither obesity nor duration of operation appeared to be associated with complications in the present exploratory analysis.

Prepectoral techniques have been introduced with the aim of improving cosmetic outcome and avoiding animation for patients electing to undergo implant-based procedures. Collection of these longer-term patient-reported outcomes is ongoing, but the present study provides reassuring data that the short-term complication rates of prepectoral and subpectoral implant reconstruction are broadly equivalent^17,22^ as the rates of implant loss, reoperation, and readmission reported here are strikingly similar to those reported in the UK iBRA study^12^, which included mainly subpectoral mesh-assisted implant-based techniques. Although complication rates are comparable between techniques in the UK setting, they are markedly higher than those reported in other recent multicentre studies of prepectoral reconstruction^58^ and in meta-analyses comparing techniques^22^. Notably, implant loss rates at 3 months in the present study are more than twice as high as the 3.1 per cent implant loss rate reported in the international iBAG study, which included 1450 prepectoral reconstructions with Braxon® (DECOmed Srl, Venice, Italy) mesh in 1186 women at 30 centres across Europe. Similarly, less than 2 per cent of patients in the iBAG study were reported to have experienced a postoperative infection compared with almost 20 per cent of women in the present analysis. Reasons for this are unclear, but iBAG was a retrospective audit that lacked prespecified definitions of outcomes, so it is possible that complications such as infection were under-reported. Furthermore, centres participating in the iBAG study were likely to be those with the most experience of the technique, so reported complication rates may not reflect wider practice. The Pre-BRA study comprises a prospective evaluation of real-world practice and outcomes in 40 UK centres and is therefore more likely to be representative of the true outcomes of the technique in this setting.

The present study has a number of limitations that require consideration. The main limitation relates to the observational study design, which introduces the risk of several forms of bias. In particular, it is possible that participating centres selectively recruited low-risk patients having prepectoral reconstruction into the study, but did not invite higher-risk patients to take part, raising the possibility of selection bias. Although, in line with guidance from the professional associations^59^, women offered implant-based reconstruction during the COVID-19 pandemic were more likely to be considered at low risk of complications than those recruited earlier in the study, overall, the proportion of smokers, those with previous chest wall radiotherapy, and women with a high BMI is remarkably consistent with that in other published UK studies^12^, suggesting that significant selection bias is unlikely. Previous chest wall radiotherapy, surgeons’ intraoperative concerns about mastectomy skin-flap viability, mastectomy weight exceeding 600 g, and smoking were associated with postoperative complications in the exploratory univariable risk factor analysis, but the study was not adequately powered to allow multivariable modelling; therefore, these findings, although compatible with the results of other studies^12,60^, should be interpreted with caution. The primary outcome of implant loss was assessed at 3 months. It is acknowledged that implant loss can occur up to 12 months following implant-based reconstruction^61^. This may represent an underestimation of the true proportion of women who ultimately experience this complication, particularly in light of the high proportion of women recommended postmastectomy radiotherapy^62^. The aim of this study, however, was to generate safety data that could be directly compared with those from the large iBRA cohort, which included mainly subpectoral reconstructions with mesh. As such, the study achieved this goal and significantly added to the evidence base for prepectoral techniques.

This study provides additional high-quality evidence to suggest that the short-term safety outcomes of prepectoral and subpectoral implant-based breast reconstruction are largely equivalent, and strongly supports the need for a definitive RCT to determine the optimal approach to implant-based reconstruction. The need for an RCT has been recognized internationally and there are currently seven RCTs currently recruiting or in set-up^63–65^. Most of these are small single-centre (NCT04716959, NCT02775409, NCT03959709) explanatory trials with tightly defined inclusion criteria (NCT04688697), limited follow-up or non-patient-centred endpoints (NCT03959709). Furthermore, two studies are from North America (NCT04716959, NCT02775409) and involve two-stage reconstruction, so do not reflect UK practice. The OPBC-02/PREPEC study^64^ is an international pragmatic RCT that is currently recruiting. This study aims to recruit 372 patients to either prepectoral or subpectoral reconstruction using any technique, with a primary outcome of change in physical function assessed using the BREAST-Q at 24 months. The UK Best-BRA study^65^ is an external pilot RCT with an embedded QuinteT Recruitment Intervention^66,67^ that will determine whether it is possible to recruit to a large-scale trial comparing prepectoral and subpectoral implant-based techniques. If the feasibility study is successful, it is anticipated that a main trial will evaluate satisfaction with breasts at 12 months using the validated BREAST-Q questionnaire^56^. Despite the variation in study designs, most RCTs are the BREAST-Q as either a primary or secondary endpoint. This will allow the results of individual studies to be combined in a meaningful meta-analysis when the trials have reported and generate further evidence to support practice.

Despite the proposed benefits of prepectoral reconstruction for patients, complication rates are high and in line with those reported for subpectoral mesh-assisted techniques. Robust evidence is required to support the benefits of this approach, and the uncertainty regarding outcomes and best practice should be discussed in detail with patients considering surgery.

## Collaborators

Members of the Pre-BRA Feasibility Study Steering Group: Peter Barry, Rachel O'Connell (The Royal Marsden NHS Foundation Trust, London, UK), Simon Cawthorn (North Bristol NHS Trust, Bristol, UK), Matthew Gardiner (Frimley Health Foundation NHS Trust and University of Oxford, Oxford, UK), Gareth Irwin (Belfast Health and Social Care Trust, Belfast, UK), Cliona Kirwan (Manchester University NHS Foundation Trust and Manchester University, UK), Mairead McKenzie (Independant Cancer Patients' Voice), Shireen McKenzie (The Leeds Teaching Hospitals NHS Trust, Leeds, UK), Georgette Oni, Lisa Whisker (Nottingham University Hospitals NHS Trust, Nottingham, UK) , Tim Rattay (University of Leicester, Leicester UK), Pankaj Roy (Oxford University Hospitals NHS Foundation Trust, Oxford, UK), Joanna Skillman (University Hospitals Coventry and Warwickshire, UK), Soni Soumian (University Hospitals of North Midlands NHS Trust, Stoke on Trent, UK), Raghavan Vidya (Royal Wolverhampton NHS Trust, Wolverhampton, UK), and Samantha Williams (Great Western Hospitals NHS Foundation Trust, Swindon, UK).

## Supplementary Material

znac077_Supplementary_Data

## Data Availability

Data are available upon reasonable request. Deidentified participant data from the safety study will be available from the senior author after completion of the study and planned analyses following review by the study steering group. Reuse will be permitted with consent of the study steering group. No additional information will be available.

## References

[1] Matala CM, McIntosh SA, Purushotham AD. Immediate breast reconstruction after mastectomy for cancer. Br J Surg 2000;87:1455–1472 11091232 10.1046/j.1365-2168.2000.01593.x

[2] Cancer Research UK . Breast Cancer Statistics. https://www.cancerresearchuk.org/health-professional/cancer-statistics/statistics-by-cancer-type/breast-cancer#heading-Zero (accessed 16 January 2022)

[3] National Institute for Health and Care Excellence (NICE) . Early and Locally Advanced Breast Cancer: Diagnosis and Management. NICE guideline [NG101]. https://www.nice.org.uk/guidance/ng101 (accessed 18 January 2022) 35263062

[4] Mennie JC, Mohanna PN, O’Donoghue JM, Rainsbury R, Cromwell DA. National trends in immediate and delayed post-mastectomy reconstruction procedures in England: a seven-year population-based cohort study. Eur J Surg Oncol 2017;43:52–61 27776942 10.1016/j.ejso.2016.09.019

[5] Albornoz CR, Bach PB, Mehrara BJ, Disa JJ, Pusic AL, McCarthy CM et al A paradigm shift in US breast reconstruction: increasing implant rates. Plast Reconstr Surg 2013;131:15–23 23271515 10.1097/PRS.0b013e3182729cde

[6] Salzberg CA, Ashikari AY, Koch RM, Chabner-Thompson E. An 8-year experience of direct-to-implant immediate breast reconstruction using human acellular dermal matrix (AlloDerm). Plast Reconstr Surg 2011;127:514–524 21285756 10.1097/PRS.0b013e318200a961

[7] Mylvaganam S, Conroy E, Williamson PR, Barnes NLP, Cutress RI, Gardiner MD et al Variation in the provision and practice of implant-based breast reconstruction in the UK: results from the iBRA national practice questionnaire. Breast 2017;35:182–190 28768227 10.1016/j.breast.2017.07.016 PMC5590633

[8] Dikmans RE, Negenborn VL, Bouman MB, Winters HA, Twisk JW, Ruhe PQ et al Two-stage implant-based breast reconstruction compared with immediate one-stage implant-based breast reconstruction augmented with an acellular dermal matrix: an open-label, phase 4, multicentre, randomised, controlled trial. Lancet Oncol 2017;18:251–258 28012977 10.1016/S1470-2045(16)30668-4

[9] Negenborn VL, Young-Afat DA, Dikmans REG, Smit JM, Winters HAH, Don Griot JPW et al Quality of life and patient satisfaction after one-stage implant-based breast reconstruction with an acellular dermal matrix *versus* two-stage breast reconstruction (BRIOS): primary outcome of a randomised, controlled trial. Lancet Oncol 2018;19:1205–1214 30104147 10.1016/S1470-2045(18)30378-4

[10] Lohmander F, Lagergren J, Roy PG, Johansson H, Brandberg Y, Eriksen C et al Implant based breast reconstruction with acellular dermal matrix: safety data from an open-label, multicenter, randomized, controlled trial in the setting of breast cancer treatment. Ann Surg 2019;269:836–841 30308615 10.1097/SLA.0000000000003054

[11] Lohmander F, Lagergren J, Johansson H, Roy PG, Frisell J, Brandberg Y. Quality of life and patient satisfaction after implant-based breast reconstruction with or without acellular dermal matrix: randomized clinical trial. BJS Open 2020;4:811–820 32762012 10.1002/bjs5.50324 PMC7528522

[12] Potter S, Conroy EJ, Cutress RI, Williamson PR, Whisker L, Thrush S et al Short-term safety outcomes of mastectomy and immediate implant-based breast reconstruction with and without mesh (iBRA): a multicentre, prospective cohort study. Lancet Oncol 2019;20:254–266 30639093 10.1016/S1470-2045(18)30781-2 PMC6358590

[13] Tasoulis MK, Iqbal FM, Cawthorn S, MacNeill F, Vidya R. Subcutaneous implant breast reconstruction: time to reconsider? Eur J Surg Oncol 2017;43:1636–1646 28528191 10.1016/j.ejso.2017.04.008

[14] Cattelani L, Polotto S, Arcuri MF, Pedrazzi G, Linguadoca C, Bonati E. One-step prepectoral breast reconstruction with dermal matrix-covered implant compared to submuscular implantation: functional and cost evaluation. Clin Breast Cancer 2018;18:e703–e711 29275104 10.1016/j.clbc.2017.11.015

[15] Sobti N, Weitzman RE, Nealon KP, Jimenez RB, Gfrerer L, Mattos D et al Evaluation of capsular contracture following immediate prepectoral *versus* subpectoral direct-to-implant breast reconstruction. Sci Rep 2020;10:1137 31980737 10.1038/s41598-020-58094-4 PMC6981172

[16] Franceschini G, Scardina L, Di Leone A, Terribile DA, Sanchez AM, Magno S et al Immediate prosthetic breast reconstruction after nipple-sparing mastectomy: traditional subpectoral technique *versus* direct-to-implant prepectoral reconstruction without acellular dermal matrix. J Pers Med 2021;11:152 33671712 10.3390/jpm11020153 PMC7926428

[17] Li L, Su Y, Xiu B, Huang X, Chi W, Hou J et al Comparison of prepectoral and subpectoral breast reconstruction after mastectomies: a systematic review and meta analysis. Eur J Surg Oncol 2019;45:1542–1550 31256950 10.1016/j.ejso.2019.05.015

[18] Snyderman RK, Guthrie RH. Reconstruction of the female breast following radical mastectomy. Plast Reconstr Surg 1971;47:565–567 5088650 10.1097/00006534-197106000-00008

[19] Gruber RP, Kahn RA, Lash H, Maser MR, Apfelberg DB, Laub DR. Breast reconstruction following mastectomy: a comparison of submuscular and subcutaneous techniques. Plast Reconstr Surg 1981;67:312–317 7232564 10.1097/00006534-198103000-00007

[20] Rebowe RE, Allred LJ, Nahabedian MY. The evolution from subcutaneous to prepectoral prosthetic breast reconstruction. Plast Reconstr Surg Glob Open 2018;6:e1797 30276046 10.1097/GOX.0000000000001797 PMC6157949

[21] Wagner RD, Braun TL, Zhu H, Winocour S. A systematic review of complications in prepectoral breast reconstruction. J Plast Reconstr Aesthet Surg 2019;72:1051–1059 31076195 10.1016/j.bjps.2019.04.005

[22] Li Y, Xu G, Yu N, Huang J, Long X. Prepectoral *versus* subpectoral implant-based breast reconstruction: a meta-analysis. Ann Plast Surg 2020;85:437–447 31913902 10.1097/SAP.0000000000002190

[23] Avila A, Bartholomew AJ, Sosin M, Deldar R, Griffith KF, Willey SC et al Acute postoperative complications in prepectoral *versus* subpectoral reconstruction following nipple-sparing mastectomy. Plast Reconstr Surg 2020;146:715e–720e 10.1097/PRS.0000000000007326 33234947

[24] Belmonte BM, Campbell CA. Safety profile and predictors of aesthetic outcomes after prepectoral breast reconstruction with meshed acellular dermal matrix. Ann Plast Surg 2021;86(Suppl 5):S585–S592 34100818 10.1097/SAP.0000000000002764

[25] Braun SE, Dreicer M, Butterworth JA, Larson KE. Do nipple necrosis rates differ in prepectoral *versus* submuscular implant-based reconstruction after nipple-sparing mastectomy? Ann Surg Oncol 2020;27:4760–4766 32699924 10.1245/s10434-020-08887-8

[26] Caputo GG, Zingaretti N, Kiprianidis I, Zanfisi C, Domenici L, Parodi PC et al Quality of life and early functional evaluation in direct-to-implant breast reconstruction after mastectomy: a comparative study between prepectoral *versus* dual-plane reconstruction. Clin Breast Cancer 2020;21:344–351 33308993 10.1016/j.clbc.2020.11.013

[27] Gabriel A, Sigalove S, Storm-Dickerson TL, Sigalove NM, Pope N, Rice J et al Dual-plane *versus* prepectoral breast reconstruction in high-body mass index patients. Plast Reconstr Surg 2020;145:1357–1365 32195862 10.1097/PRS.0000000000006840

[28] Kim JH, Hong SE. A comparative analysis between subpectoral *versus* prepectoral single stage direct-to-implant breast reconstruction. Medicina 2020;56:537 10.3390/medicina56100537 PMC7602109 33066236

[29] King CA, Bartholomew AJ, Sosin M, Avila A, Famiglietti AL, Dekker PK et al A critical appraisal of late complications of prepectoral *versus* subpectoral breast reconstruction following nipple-sparing mastectomy. Ann Surg Oncol 2021;28:9150–9158 34386913 10.1245/s10434-021-10085-z

[30] Manrique OJ, Banuelos J, Abu-Ghname A, Nguyen MD, Tran NV, Martinez-Jorge J et al Surgical outcomes of prepectoral *versus* subpectoral implant-based breast reconstruction in young women. Plast Reconstr Surg Glob Open 2019;7:e2119 31044105 10.1097/GOX.0000000000002119 PMC6467633

[31] Mirhaidari SJ, Azouz V, Wagner DS. Prepectoral *versus* subpectoral direct to implant immediate breast reconstruction. Ann Plast Surg 2020;84:263–270 31663934 10.1097/SAP.0000000000002059

[32] Momeni A, Remington AC, Wan DC, Nguyen D, Gurtner GC. A matched-pair analysis of prepectoral with subpectoral breast reconstruction: is there a difference in postoperative complication rate? Plast Reconstr Surg 2019;144:801–807 31568276 10.1097/PRS.0000000000006008

[33] Ng EEI, Quah GS, Graham S, Kanesalingam K, Meybodi F, Hsu J et al Immediate prepectoral implant reconstruction using TiLOOP Bra Pocket results in improved patient satisfaction over dual plane reconstruction. ANZ J Surg 2021;91:701–707 33634944 10.1111/ans.16670

[34] Thangarajah F, Treeter T, Krug B, Hellmich M, Eichler C, Hanstein B et al Comparison of subpectoral *versus* prepectoral immediate implant reconstruction after skin- and nipple-sparing mastectomy in breast cancer patients: a retrospective hospital-based cohort study. Breast Care 2019;14:382–387 31933584 10.1159/000496696 PMC6940459

[35] Viezel-Mathieu A, Alnaif N, Aljerian A, Safran T, Brabant G, Boileau JF et al Acellular dermal matrix-sparing direct-to-implant prepectoral breast reconstruction: a comparative study including cost analysis. Ann Plast Surg 2020;84:139–143 31335468 10.1097/SAP.0000000000001997

[36] Thuman JM, Worbowtiz N, Jain A, Ulm JP, Delaney KO, Herrera FA. Impact of radiation on implant-based breast reconstruction in prepectoral *versus* submuscular planes. Ann Plast Surg 2021;86(Suppl 5):S560–S566 34100813 10.1097/SAP.0000000000002882

[37] Ribuffo D, Berna G, De Vita R, Di Benedetto G, Cigna E, Greco M et al Dual-plane retro-pectoral *versus* pre-pectoral DTI breast reconstruction: an Italian multicenter experience. Aesthetic Plast Surg 2021;45:51–60 32860077 10.1007/s00266-020-01892-y PMC7886728

[38] Walker NJ, Park JG, Maus JC, Motamedi V, Rebowe RE, Runyan CM et al Prepectoral *versus* subpectoral breast reconstruction in high-body mass index patients. Ann Plast Surg 2021;87:136–143 33560000 10.1097/SAP.0000000000002682

[39] Bozzuto LM, Bartholomew AJ, Tung S, Sosin M, Tambar S, Cox S et al Decreased postoperative pain and opioid use following prepectoral *versus* subpectoral breast reconstruction after mastectomy: a retrospective cohort study: pain after pre- *versus* subpectoral reconstruction. J Plast Reconstr Aesthet Surg 2021;74:1763–1769 33451949 10.1016/j.bjps.2020.12.009

[40] Copeland-Halperin LR, Yemc L, Emery E, Collins D, Liu C, Mesbahi AN et al Evaluating postoperative narcotic use in prepectoral *versus* dual-plane breast reconstruction following mastectomy. Plast Reconstr Surg Glob Open 2019;7:e2082 30881831 10.1097/GOX.0000000000002082 PMC6416120

[41] Lee JS, Park E, Lee JH, Lee J, Park HY, Yang JD et al A prospective comparison study of early functional outcomes after implant-based breast reconstruction: subpectoral *versus* prepectoral technique. Ann Palliat Med 2021;10:2520–2529 33691448 10.21037/apm-20-1550

[42] Wormer BA, Valmadrid AC, Ganesh Kumar N, Al Kassis S, Rankin TM, Kaoutzanis C et al Reducing expansion visits in immediate implant-based breast reconstruction: a comparative study of prepectoral and subpectoral expander placement. Plast Reconstr Surg 2019;144:276–286 31348326 10.1097/PRS.0000000000005791

[43] Sewart E, Turner NL, Conroy EJ, Cutress RI, Skillman J, Whisker L et al Patient-reported outcomes of immediate implant-based breast reconstruction with and without biological or synthetic mesh. BJS Open 2021;5. DOI:10.1093/bjsopen/zraa063 PMC7896806 33609398

[44] Potter S, Mills N, Cawthorn SJ, Donovan J, Blazeby JM. Time to be BRAVE: is educating surgeons the key to unlocking the potential of randomised clinical trials in surgery? A qualitative study. Trials 2014;15:80 24628821 10.1186/1745-6215-15-80 PMC4003809

[45] Winters ZE, Emson M, Griffin C, Mills J, Hopwood P, Bidad N et al Learning from the QUEST multicentre feasibility randomization trials in breast reconstruction after mastectomy. Br J Surg 2015;102:45–56 25451179 10.1002/bjs.9690

[46] Ergina PL, Cook JA, Blazeby JM, Boutron I, Clavien PA, Reeves BC et al Challenges in evaluating surgical innovation. Lancet 2009;374:1097–1104 19782875 10.1016/S0140-6736(09)61086-2 PMC2855679

[47] Pennell CP, Hirst AD, Campbell WB, Sood A, Agha RA, Barkun JST et al Practical guide to the Idea. Development and Exploration stages of the IDEAL Framework and Recommendations. Br J Surg 2016;103:607–615 26865013 10.1002/bjs.10115

[48] Harvey KL, Mills N, White P, Holcombe C, Potter S. The Pre-BRA (pre-pectoral breast reconstruction EvAluation) feasibility study: protocol for a mixed-methods IDEAL 2a/2b prospective cohort study to determine the safety and effectiveness of prepectoral implant-based breast reconstruction. BMJ Open 2020;10:e033641 10.1136/bmjopen-2019-033641 PMC7044855 31988232

[49] Martin L, O’Donoghue JM, Horgan K, Thrush S, Johnson R, Gandhi A et al Acellular dermal matrix (ADM) assisted breast reconstruction procedures: joint guidelines from the Association of Breast Surgery and the British Association of Plastic, Reconstructive and Aesthetic Surgeons. Eur J Surg Oncol 2013;39:425–429 23321393 10.1016/j.ejso.2012.12.012

[50] Harris PA, Taylor R, Thielke R, Payne J, Gonzalez N, Conde JG. Research electronic data capture (REDCap)—a metadata-driven methodology and workflow process for providing translational research informatics support. J Biomed Inform 2009;42:377–381 18929686 10.1016/j.jbi.2008.08.010 PMC2700030

[51] Blencowe NS, Mills N, Cook JA, Donovan JL, Rogers CA, Whiting P et al Standardizing and monitoring the delivery of surgical interventions in randomized clinical trials. Br J Surg 2016;103:1377–1384 27462835 10.1002/bjs.10254 PMC5132147

[52] Rainsbury D, Willett A. Oncoplastic Breast Reconstruction: Guidelines for Best Practice. London: Royal College of Surgeons of England, 2012

[53] Barr SP, Topps AR, Barnes NL, Henderson J, Hignett S, Teasdale RL et al Infection prevention in breast implant surgery—a review of the surgical evidence, guidelines and a checklist. Eur J Surg Oncol 2016;42:591–603 27005885 10.1016/j.ejso.2016.02.240

[54] O’Connell RL, Rattay T, Dave RV, Trickey A, Skillman J, Barnes NLP et al The impact of immediate breast reconstruction on the time to delivery of adjuvant therapy: the iBRA-2 study. Br J Cancer 2019;120:883–895 30923359 10.1038/s41416-019-0438-1 PMC6734656

[55] Potter S, Davies C, Holcombe C, Weiler-Mithoff E, Skillman J, Vidya R et al International development and implementation of a core measurement set for research and audit studies in implant-based breast reconstruction: a study protocol. BMJ Open 2020;10:e035505 10.1136/bmjopen-2019-035505 PMC7045234 31964677

[56] Pusic AL, Klassen A, Scott AF, Klok JA, Cordeiro PG, Cano S. Development of a new patient-reported outcome measure for breast surgery: the BREAST-Q. Plast Reconstr Surg 2009;124:345–353 19644246 10.1097/PRS.0b013e3181aee807

[57] Dave RV, Kim B, Courtney A, O’Connell R, Rattay T, Taxiarchi VP et al Breast cancer management pathways during the COVID-19 pandemic: outcomes from the UK ‘Alert Level 4’ phase of the B-MaP-C study. Br J Cancer 2021;124:1785–1794 33767422 10.1038/s41416-020-01234-4 PMC7993073

[58] Masià J . The largest multicentre data collection on prepectoral breast reconstruction: the iBAG study. J Surg Oncol 2020;122:848–860 32786089 10.1002/jso.26073 PMC7540676

[59] Association of Breast Surgery . Moving Forward—Recommendations from the Association of Breast Surgery on Delivery of Breast Services During the Covid-190 Pandemic. https://associationofbreastsurgery.org.uk/media/252009/abs-statement-150320-v2.pdf (accessed 25 May 2020)

[60] Knight HJ, Musgrove JJ, Youssef MMG, Ferguson DJ, Olsen SB, Tillett RL. Significantly reducing implant loss rates in immediate implant-based breast reconstruction: a protocol and completed audit of quality assurance. J Plast Reconstr Aesthet Surg 2020;73:1043–1049 32008945 10.1016/j.bjps.2019.12.005

[61] Blough JT, Vu MM, Qiu CS, Mlodinow AS, Khavanin N, Fine NA et al Beyond 30 days: a risk calculator for longer term outcomes of prosthetic breast reconstruction. Plast Reconstr Surg Glob Open 2018;6:e2065 30656128 10.1097/GOX.0000000000002065 PMC6326616

[62] Jagsi R, Momoh AO, Qi J, Hamill JB, Billig J, Kim HM et al Impact of radiotherapy on complications and patient-reported outcomes after breast reconstruction. J Natl Cancer Inst 2018;110:157–165 28954300 10.1093/jnci/djx148 PMC6059091

[63] Dyrberg DL, Gunnarsson GL, Bille C, Sørensen JA, Thomsen JB. Direct-to-implant extracellular matrix hammock-based breast reconstruction; prepectoral or subpectoral? Trials 2020;21:160 32041661 10.1186/s13063-020-4125-6 PMC7011213

[64] Kappos EA, Schulz A, Regan MM, Moffa G, Harder Y, Ribi K et al Prepectoral *versus* subpectoral implant-based breast reconstruction after skin-sparing mastectomy or nipple-sparing mastectomy (OPBC-02/PREPEC): a pragmatic, multicentre, randomised, superiority trial. BMJ Open 2021;11:e045239 10.1136/bmjopen-2020-045239 PMC8413865 34475143

[65] Roberts K, Mills N, Metcalfe C, Lane A, Clement C, Hollingworth W et al Best-BRA (Is subpectoral or pre-pectoral implant placement best in immediate breast reconstruction?) A protocol for a pilot randomised controlled trial of subpectoral *versus* pre-pectoral immediate implant-based breast reconstruction in women following mastectomy. BMJ Open 2021;11:e050886 10.1136/bmjopen-2021-050886 PMC8634330 34848516

[66] Rooshenas L, Scott LJ, Blazeby JM, Rogers CA, Tilling KM, Husbands S et al The QuinteT recruitment intervention supported five randomized trials to recruit to target: a mixed-methods evaluation. J Clin Epidemiol 2019;106:108–120 30339938 10.1016/j.jclinepi.2018.10.004 PMC6355457

[67] Donovan JL, Rooshenas L, Jepson M, Elliott D, Wade J, Avery K et al Optimising recruitment and informed consent in randomised controlled trials: the development and implementation of the Quintet Recruitment Intervention (QRI). Trials 2016;17:283 27278130 10.1186/s13063-016-1391-4 PMC4898358

