# Supplementary material for: Short-term safety outcomes of mastectomy and immediate prepectoral implant-based breast reconstruction: Pre-BRA prospective multicentre cohort study
Source: Br J Surg. 2022 Apr 5;109(6):530–8. doi: 10.1093/bjs/znac077 (PMC10364707; doi:10.1093/bjs/znac077)
Supplement: znac077_Supplementary_Data [file znac077_supplementary_data.zip › Supplementary_material.docx]

**Table S1. Surgical data**

| **Per breast data** | **N=424 (%)** |
| --- | --- |
| **Planned procedure**  Planned one-stage  Planned two-stage  Not reported | 357 (84.2)  60 (14.2)  7 (1.7) |
| **Grade of operating surgeon**  Consultant  Staff grade/Associate Specialist  Senior Trainee (OPF/ST8+)  Junior trainee (ST6 or below)  Missing | 362 (85.4)  23 (5.4)  30 (7.1)  8 (1.9)  1 (0.2) |
| **Total cases performed by operating surgeon (supervised and unsupervised)**  <5  5-10  11-25  >25  Not reported | 28 (6.6)  55 (13.0)  88 (20.8)  241 (56.8)  12 (2.8) |
| **Operations performed unsupervised**  <5  5-10  11-25  >25  Not reported | 51 (12.0)  54 (12.7)  67 (15.8)  189 (44.6)  63 (14.9) |
| **Type of mastectomy**  Skin sparing  Nipple sparing  Reduction (wise) pattern  Other  Not reported | 134 (31.6)  221 (52.1)  64 (15.1)  4 (0.9)  1 (0.2) |
| **Incision**  Periareolar (nipple sparing)  Lateral  Inframammary  Elliptical removing NAC  Wise pattern  Other  Not reported | 30 (7.1)  64 (15.1)  119 (28.1)  112 (26.4)  71 (16.8)  24 (5.7)  4 (0.9) |
| **Hydro dissection of mastectomy plane**  Not used  With saline  With local anaesthetic (LA) +/- saline  With LA + adrenaline +/- saline  Not reported | 301 (71.0)  19 (4.5)  26 (6.1)  73 (17.2)  5 (1.2) |
| **Main instrument used for dissection of mastectomy skin flaps**  Blade  Scissors  Hand-held monopolar diathermy  Diathermy scissors  Combination of methods  Other  Not reported | 36 (8.5)  82 (19.3)  250 (59.0)  23 (5.4)  28 (6.6)  1 (0.2)  4 (0.9) |
| **Mastectomy weight (median, IQR, range)** | 410 (263-590)  (49-2009) |
| **Surgeon’s assessment of skin flap quality**  Good – no concerns  Average – no obvious concerns at time of surgery  Poor – thin flaps, questionable vascularity  Not reported | 330 (77.8)  81 (19.1)  9 (2.1)  4 (0.9) |
| **Was the procedure performed as planned?**  Yes  No  Not reported | 411 (96.9)  8 (1.9)  5 (1.2) |
| **Actual procedure performed**  PPBR considered safe and performed with fixed volume implant  PPBR performed with adjustable implant (e.g. Becker)  PPBR performed with tissue expander  PPBR abandoned*  Not reported | 288 (67.9)  43 (10.1)  30 (7.1)  4 (0.9)  59 (13.9) |
| **Type of implant coverage**  Subcutaneous only (no mesh used)  Biological mesh  Synthetic mesh  Complete dermal sling  Dermal sling/biological mesh in combination  Dermal sling/synthetic mesh in combination  Other  Not reported | 4 (0.9)  341 (80.4)  32 (7.6)  1 (0.2)  38 (9.0)  4 (0.9)  1 (0.2)  3 (0.7) |
| **Type of biological mesh used (n=377)**  BRAXON  SurgiMend  Strattice  MESO  Fortiva  Exaflex pocket  Other | 196 (52.0)  129 (34.2)  11 (2.9)  10 (2.7)  15 (4.0)  4 (1.1)  13 (3.4) |
| **Synthetic mesh type (n=36)**  TiLOOP  Other | 35 (97.2)  1 (2.8) |
| **Breast prosthesis used**  Fixed volume implant  Temporary tissue expander  Adjustable implant (e.g. Beckers)  Missing | 334 (78.8)  44 (10.4)  45 (10.6)  1 (0.2) |
| **Implant size (cc) (median, IQR, range)** | 395 (300-480)  (125-835) |
| **Implant shape**  Anatomical  Round  Not reported | 347 (81.8)  71 (16.8)  6 (1.4) |
| **Implant texture**  Textured/microtextured  Smooth  Polyurethane  Not reported | 366 (86.3)  39 (9.2)  6 (1.4)  13 (3.1) |
| **Skin edges excised before closure** | 161 (38.0) |
| **Axillary surgery**  None  Sentinel lymph node biopsy  Axillary sample  Axillary node clearance  SNB + ALND  Not reported | 161 (38.0)  211 (49.8)  9 (2.1)  36 (8.5)  5 (1.2)  2 (0.5) |
| **Number of drains used per breast**  None  One  Two  Not reported | 4 (0.9)  291 (68.6)  124 (29.3)  5 (1.2) |
| **Dressings used^a^**  Occlusive dressing  Skin glue  Skin closure system  PICO  Other | 261 (61.6)  127 (30.0)  24 (5.7)  116 (27.4)  16 (3.8) |
| **Per patient perioperative data** | **N=347** |
| **Antibiotics used**  Prophylactic only (<24 hours)  1-5 days  Extended course (%+ days)  Until drains out  Other  Not reported | 56 (16.1)  71 (20.5)  63 (18.2)  150 (43.2)  3 (0.9)  4 (1.2) |
| **Duration of Procedure (knife to skin to dressing on) (mins) (IQR) (range)** | 165.5 (130-190.5)  (60-420) |
| **Length of stay**  Daycase  Overnight stay  2 nights  More than 2 nights  Not reported | 50 (14.4)  200 (57.6)  54 (15.5)  35 (10.1)  8 (2.3) |

IQR – interquartile range; OPF – oncoplastic fellow; PPBR – pre-pectoral breast reconstruction; ST – speciality trainee, SNB sentinel node bipsy; ALND axillary lymph node dissection

^a^Patients may have had more than one type of dressing applied

*All four cases in whom PPBR was abandoned, three were due to thin skin flaps and one due to two previous wide local excisions; of these all had subpectoral implant-based reconstruction

**Table S2. Details of infection prevention measures used in Pre-BRA study**

|  | **N=347 (%)** |
| --- | --- |
| **Infection prevention measures**  Laminar flow  Yes  No  Not reported | 166 (47.8)  170 (49.0)  11 (3.2) |
| **Skin preparation used**  Iodine  Chlorhexidine  2% Cholorprep  Other  Not reported | 97 (28.0)  122 (35.2)  117 (33.7)  2 (0.6)  9 (2.6) |
| **Antibiotics used**  Prophylactic only (<24 hours)  1-5 days  Extended course (%+ days)  Until drains out  Other  Not reported | 56 (16.1)  71 (20.5)  63 (18.2)  150 (43.2)  3 (0.9)  4 (1.2) |
| **Antibiotics most commonly used**  Co-amoxiclav  Flucloxacillin  Gentamicin  Teicoplanin | 162 (46.7)  76 (21.9)  120 (34.6)  76 (21.9) |
| **Antibiotics at induction**  Yes  Not reported | 342 (98.6)  5 (1.4) |
| **Patient warming**  Yes  No  Not reported | 337 (97.1)  3 (0.9)  7 (1.0) |
| ‘**No entry’ signs on theatre doors**  Yes  No  Not reported | 289 (83.3)  51 (14.7)  7 (2.0) |
| **Surgeons double gloved**  Yes  No  Not reported | 246 (70.9)  94 (27.1)  7 (2.0) |
| **Alcoholic skin prep used**  Yes  No  Not reported | 290 (83.6)  50 (14.4)  7 (2.0) |
| **Implant pocket washed out**  Yes  No  Not reported | 321 (92.5)  20 (5.8)  6 (1.7) |
| **Surgeon glove change before handling implant**  Yes  No  Not reported | 333 (96.0)  4 (1.2)  10 (2.9) |
| **Use of a tunnelled drain**  Yes  No  Not reported | 331 (95.4)  9 (2.6)  7 (2.0) |
| **Post-operative antibiotics prescribed for patients considered high risk**  Yes  No  Not reported | 296 (85.3)  40 (11.5)  11 (3.2) |

**Table S3. Details of complications at three months**

| **Complications** | **N (%)**  **(95% Confidence interval)** | **Classification of complication** | **N=343 (%)** |
| --- | --- | --- | --- |
| **Any complication** | 144 (42.0)  (36.6-47.1) | Requiring readmission  Requiring re-operation | 60 (17.5)  55 (16.0) |
| **Implant loss** | 28 (8.2)  (5.3-11.1) | Total implant loss  Successful implant salvage | 28 (8.1)  11 (3.2) |
| **Infection** | 67 (19.5)  (15.3-23.8) | Requiring oral antibiotics  Requiring admission for IV antibiotics  Requiring surgical drainage/debridement  No additional details reported | 27 (40.3)  8 (11.9)  27 (40.3)  5 (7.5) |
| **Seroma** | 71 (20.7)  (16.9-25.7) | Not requiring aspiration  Requiring aspiration 1-2 times  Requiring aspiration 3 or more times  No additional details reported | 27 (38.0)  33 (46.5)  7 (9.9)  4 (5.6) |
| **Haematoma** | 5 (1.5)  (0.2-2.8) | Managed conservatively  Requiring surgical evacuation | 2 (40.0)  3 (60.0) |
| **Mastectomy skin flap necrosis** | 22 (6.6)  (3.9-9.3) | Managed conservatively  Requiring surgical debridement in clinic (no GA)  Requiring surgical debridement in theatre | 6 (27.3)  2 (9.1)  14 (63.6) |
| **Nipple necrosis (n=332)** | 18 (5.4)  (3.0-7.9) | Managed conservatively  Requiring surgical debridement  Total NAC loss  No additional details reported | 9 (50.0)  4 (22.2)  4 (22.2)  1 (6.6) |
| **Wound dehiscence (n=333)** | 16 (4.8)  (2.5-7.1) | Managed conservatively  Requiring return to theatre for re-suturing  No additional details reported | 2 (12.5)  12 (75.0)  2 (12.5) |
